# Carotenoids and Their Biosynthesis in Fungi

**DOI:** 10.3390/molecules27041431

**Published:** 2022-02-21

**Authors:** Gerhard Sandmann

**Affiliations:** Biosynthesis Group, Institute for Molecular Biosciences, Fachbereich Biowissenschaften, Goethe Universität Frankfurt, D-60438 Frankfurt, Germany; sandmann@bio.uni-frankfurt.de

**Keywords:** carotenogenic pathways, carotenoid biosynthesis, carotenoid distribution, carotenoid pathway engineering, reaction mechanisms, trisporic acids

## Abstract

Carotenoids represent a class of pigmented terpenoids. They are distributed in all taxonomic groups of fungi. Most of the fungal carotenoids differ in their chemical structures to those from other organisms. The general function of carotenoids in heterotrophic organisms is protection as antioxidants against reactive oxygen species generated by photosensitized reactions. Furthermore, carotenoids are metabolized to apocarotenoids by oxidative cleavage. This review presents the current knowledge on fungal-specific carotenoids, their occurrence in different taxonomic groups, and their biosynthesis and conversion into trisporic acids. The outline of the different pathways was focused on the reactions and genes involved in not only the known pathways, but also suggested the possible mechanisms of reactions, which may occur in several non-characterized pathways in different fungi. Finally, efforts and strategies for genetic engineering to enhance or establish pathways for the production of various carotenoids in carotenogenic or non-carotenogenic yeasts were highlighted, addressing the most-advanced producers of each engineered yeast, which offered the highest biotechnological potentials as production systems.

## 1. Introduction

Carotenoids are terpenoid pigments of yellow, orange, and red color. They are synthesized in species from all taxa with the exception of animals [1]. In contrast to photoautotrophic organism, carotenoids are not essential for fungi and are therefore accumulated in much lower concentrations than by plants and algae. However, fungal carotenoids are different to those found elsewhere, offering a structural diversity. In order to increase the levels of commercially interesting carotenoid in different fungal species and to match the concentrations of other organisms, classical and metabolic engineering procedures were established, which will be referred to. Carotenoids are found in all fungal groups along with non-carotenogenic species [2,3]. A general feature of carotenoids is their function as antioxidants. They are able to inactivate oxygen radicals and to quench singlet oxygen [4]. This reactive oxygen species is generated by photosensitized reactions. Protection from cell death caused by light and UV radiation were demonstrated with *Microbotryum violaceum* (formerly *Ustilago violacea*) and *Neurospora crassa* [5,6]. In several fungi, the synthesis of carotenoids is photo-regulated [7]. This light-dependent up-regulation corroborates the importance of antioxidative carotenoids. Another substantial role of carotenoids is their participation in the mating of mucoraceous fungi [8]. More details on the biological functions of carotenoids can be found in reference [9].

First attempts to elucidate the biosynthesis pathway were with mucoraceous fungi. Radioactivity incorporated by feeding acetate and mevalonate, both ^14^C-labelled at different positions [10], were recovered at individual positions of the β-carotene molecule [11]. The resulting labelling pattern were the first indication leading to the understanding of the reaction sequences in carotenoid biosynthesis. The establishment of the pathway was further substantiated by in vitro incorporation of mevalonate along the carotenoid pathway [12] and with different pigment mutants [13].

The review provides an updated knowledge of developments in the fungal carotenoid field. The focus is on the carotenoid structures found in fungi, their biosynthesis pathways, and considerations on the reaction mechanisms for insertion of oxy groups. This also includes distribution of carotenoids within the different fungal groups. Furthermore, the potential of fungi for the production of commercially interesting carotenoids, including their formation by pathway engineering of yeasts, was addressed.

## 2. Evolutionary Origin of Fungal Carotenoid Biosynthesis

As part of the Archaea-Eukarya lineage [14], fungi inherited the potential of carotenoid biosynthesis from archaea. Therefore, carotenoids can be found in species of all fungal phyla. Common to all fungal groups are species which form β-carotene [1,2] resembling the C40-pathway in the class of Halobacteria [15]. In addition, modifications by oxygenation of β-carotene and its precursors specifically evolved in fungi. Typical for fungal carotenogenesis is the presence of the *crtYB* fusion gene [16]. It evolved from the co-transcribed overlapping genes of *crtY* and *crtB* in Archaea where they are organized in a gene cluster, as exemplified for *Sulfolobus solfaticus* [17]. Due to the evolutionary relationship with Archaea, fungi inherited the mevalonate pathway from acetyl-CoA for carotenoid precursor synthesis [18] and are devoid of the alternative deoxyxylulose 5-phosphate pathway, which dominates in bacteria [19]. In contrast to the bacterial-chloroplast lineage, fungi did not acquire the formation of an ε-ionone ring, epoxy groups, or 3-hydroxy-β-ionone end groups (as e.g., in zeaxanthin), with the exception of the astaxanthin pathway in *Xanthophyllomyces dendrorhous* (see below). In several fungi, carotenoid synthesis is photo-regulated. Transcriptional regulation of carotenoid pathways by light and other factors in *Fusarium* (formerly *Gibberella*) *fujikuroi*, *N. crassa* [9], and *Phycomyces blakesleanus* [20] were reviewed.

Along the eukaryotic lineage, animals lost the potential of carotenoid biosynthesis. However, in aphids, such as varieties of *Acyrthosiphon pisum*, the synthesis of torulene, which is a typical fungal carotenoid, was discovered [21]. Since the *crtYB* gene, which can be regarded as a fungal signature, is present and involved in aphid carotenoid biosynthesis, it is obvious that this pathway was acquired by horizontal gene transfer from fungi.

### 2.1. The Basic Pathway to β-Carotene

**Figure 1:**
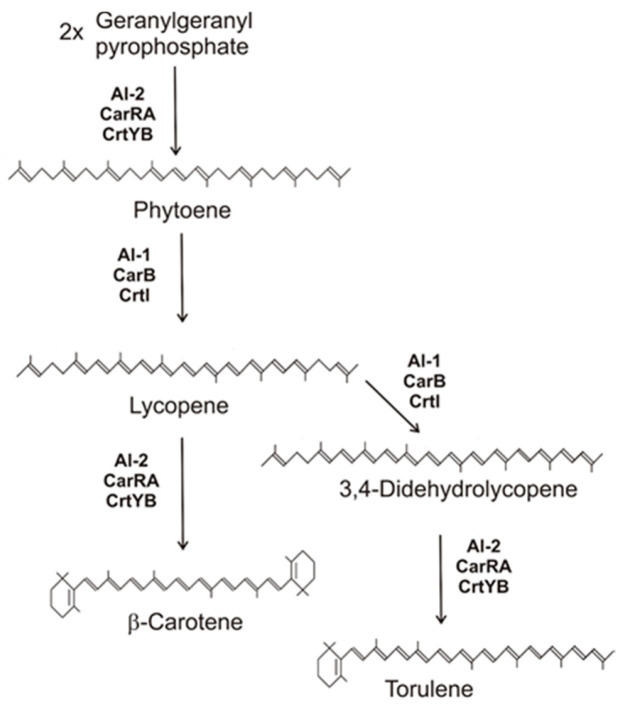
Carotenoid biosynthesis pathway of fungi to β-carotene and torulene with gene products involved.

In β-carotene synthesizing fungi, the pathway proceeds in three steps (Figure 1). After the condensation of two molecules of geranylgeranyl pyrophosphate to phytoene, a single enzyme catalyzed four desaturation steps. Finally, both enzymes were cyclized to β-ionone groups. Only two genes encode the enzymes involved: a phytoene synthase lycopene-cyclase fusion gene assigned as *al-2*, *crtYB*, or *carRA* [16,22,23] depending on the fungal species, and a phytoene desaturase gene assigned as *al-1*, *crtI*, or *carB* [23,24,25]. The *al-2* gene was first cloned from *N. crassa* as a phytoene synthase gene. For the homolog *crtYB* from *X. dendrorhous*, functional analysis revealed that the expressed gene product catalyzed not only phytoene synthesis, but also lycopene cyclization. Both functional domains could be located in the gene sequence [16]. Both domains were heterologously expressed, and antisera were raised against both proteins. With their help, an individual soluble phytoene synthase domain and an individual membrane-bound lycopene cyclase were detected in *Blakeslea trispora*, indicating a posttranslational protein cleavage [26]. The first fungal phytoene desaturase gene *al-1* was also cloned from *N. crassa* [24]. Depending on the classification of the carotenogenic species, the desaturases differ in their product-specificity. Corresponding to the synthesis of β-carotene in fungi from Mucorales, *carB* from *P. blakesleanus* encodes a 4-step desaturase which produces lycopene, as demonstrated by heterologous expression [27] and by lycopene accumulation in the mutant C9 [13]. In contrast, the gene product *al-1* from *N. crassa* inserts a fifth double bond at C3-C4 [28]. This prevents the cyclisation of this further desaturated end of the molecule, and only a single cyclisation step to torulene is possible (Figure 1 right branch).

### 2.2. Synthesis of Neurosporaxanthin in Sordariomycetes

**Figure 2:**
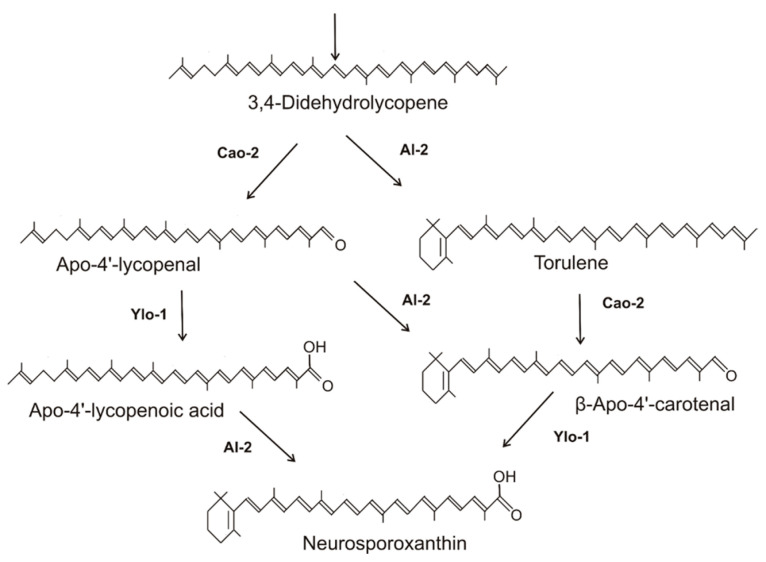
Late steps to neurosporaxanthin in *Neurospora crassa*, with corresponding fungal gene products.

The end product of carotenoid synthesis of Sordariomycetes is neurosporaxanthin. Its structure was elucidated from *N. crassa* as 4′-apo-β,ψ-caroten-4′-oate [29]. Other accumulating intermediates include 3,4-didehydrolycopene and γ-carotene [30,31]. The subsequent pathway steps leading to this C35-apocarotenoid were established for *N. crassa* based on the genes involved and the carotenoid composition of the yellow YLO mutants. Carotenoids in different YLO strains were identified as apo-4′-lycopenal and β-apo-4′- carotenal [32] or their derivatives, which were reduced and esterified with fatty acids for better sequestration [31]. These carotenoids in the YLO mutants are the products of the cleavage of the C3-C4 double bond either of 3,4-didehydrolycopene or torulene. Consequently, alternative reaction sequences involved in the late steps to neurosporaxanthin were proposed [31,32]. In carotenoid biosynthesis, enzymes recognize only a certain specific part of the molecule for catalysis. Therefore, different successions of reactions can result in a complex pathway pattern. Based on this and on the data from the above publications, a combined pathway for the late steps in neurosporaxanthin synthesis is presented in Figure 2. The three enzymes involved in parallel reaction sequences are a cyclase recognizing as substrate the ψ-end of a carotenoid, a cleavage enzyme splitting the C3-C4 double bond and a C4′-aldehyde oxidase. Starting with the cleavage reactions of 3,4-didehydrolycopene to apo-4′-lycopenal, this aldehyde is differently metabolized either by cyclization to β-apo-4′-carotenal or hydroxylation to β-apo-4′-carotenoic acid. As illustrated in Figure 2, left branch, the apo-4′-lycopenoic acid is cyclized to neurosporaxanthin. Alternatively, 3,4-didehydrolycopene was cyclized to torulene, which was also a substrate for the cleavage reaction (Figure 2, right branch). This directly resulted in the formation of β-apo-4′-carotenal, which was further hydroxylated to neurosporaxanthin. Genes involved in these late steps specific to Sordariomycetes are the cleavage enzyme encoded by the *cao-2* gene of *N. crassa* [33], also assigned as *carT* in the genus *Fusarium* [34], and the aldehyde dehydrogenase gene *ylo-1* from *N. crassa* [32] (as *carD* in *Fusarium*) [35]. For the neurosporaxanthin pathways of *F. fujikuroi* [23], *Gibberella zeae* [36], and *Podospora anserina* [37], only the branch via torulene cleavage to β-apo-4′-carotenal (Figure 2a, right branch) has been recognized.

### 2.3. Synthesis of Astaxanthin in Xanthophyllomyces denrorhous

**Figure 3:**
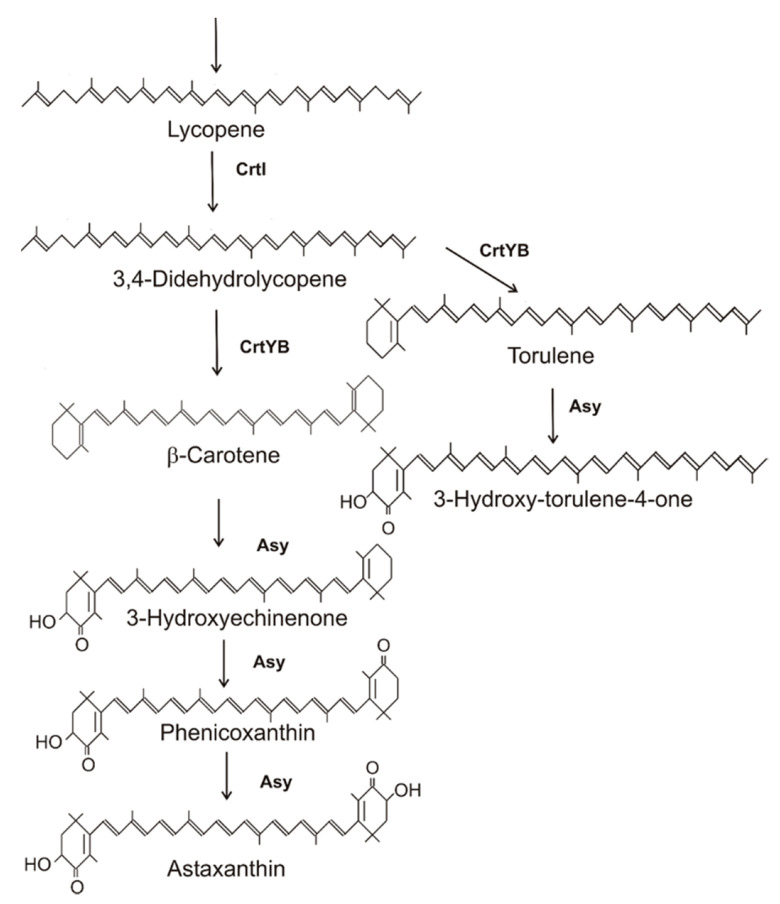
Biosynthesis pathway and gene products for astaxanthin biosynthesis in *Xanthophyllomyces dendrorhous*.

The basidiomycetous yeast *X. denrorhous* (anamorph *Phaffia rhodozyma*) is the only fungus which is able to synthesize the 3,3′-dihydroxy-4,4′-diketo β-carotene derivative astaxanthin [38]. Astaxanthin is accumulated as the 3,3′R enantiomer in contrast to the 3,3′S enantiomer synthesized by bacteria [39]. As outlined in Figure 1, the pathway to β-carotene is catalyzed by the genes indicated in this figure. The complete modification of β-carotene to astaxanthin, as illustrated in Figure 3, is catalyzed by the product of the *asy* gene [40], also named *crtS* in another publication [41]. This unique gene is absent in other organisms [40]. The encoded astaxanthin synthase is a P450 monooxygenase that interacts with the *crtR* encoded cytochrome P450 reductase [42]. The reaction mechanism of β-carotene modification by this P450 enzyme involved a series of hydroxylation steps at each β-ionone ring, forming a keto group after water elimination twice at C4, and then followed by hydroxylation of C3 [40]. The same reactions proceed at C3′ and C4′ at the other end. All the carotenoids formed by these hydroxylation reactions, the mono-ketolated echinenone, its 3-hydroxy derivative, and phoenicoxanthin which additionally carries a C4′ keto group could be identified as intermediates [38]. Another minor carotenoid of *X. denrorhous* is 3-HO-4-ketotorulene. Its synthesis diverts from the main pathway by a fifth desaturation of lycopene to 3,4,-didehydrolycopene in competition to lycopene cyclization (Figure 3, right branch). The concentration of 3-HO-4-ketotorulene below 1% of total carotenoids in *X. dendrorhous* [38] may indicate a substantial lower affinity of the desaturase than the cyclase for lycopene. In addition, metabolic engineering also showed that the ratio of lycopene cyclase to phytoene desaturase determined the ratio of β-carotene versus 3,4,-didehydrolycopene formation [43]. Changes in the gene expression of these competing enzymes may be the reason why different physiological conditions can increase the flux into the monocyclic branch [44]. Finally, 3,4,-didehydrolycopene was cyclized to torulene, into which astaxanthin synthase inserts 3-hydroxy and the 4-keto groups (Figure 3, right branch).

## 3. Formation of Carotenoid Structures and Their Distribution in Fungi

**Figure 4:**
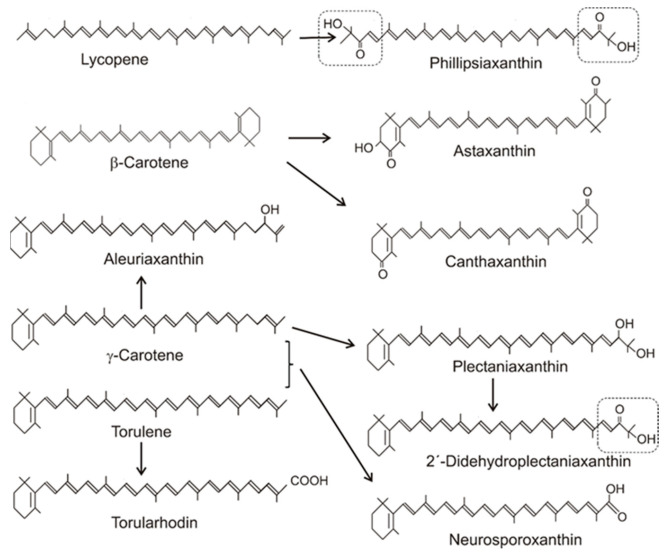
Precursors and structures of oxygenated fungal carotenoids. Identical 3′4′-didehydro-1′,2′-dihydro-1′-hydroxy-2′-one-ψ-end groups of different carotenoids are boxed.

Table 1 gives an overview on the distribution of carotenoids in different fungal groups. Most of the carotenoids of this table were only structurally analyzed and identified without the determination of their concentrations. Only for a few species, the concentrations of their specific carotenoids were quantified. Their values range from 100 to 200 µg/g dw or were even lower [20,31,45,46,47]. The accumulating carotenoid in lower fungi, in the Chytridiomycota and Blastocladiomycota, is the monocyclic γ-carotene in Table 1. Its synthesis was due to a modified lycopene cyclase which catalyzed formation of only one ionone ring instead of two, as in the case of β-carotene formation. The bicyclic β-carotene is typical for species of the Mucoraceae. It was also found as the end product in a few other fungi of Ascomycota and Basidiomycota, but most carotenoids in both phyla were oxygenated derivatives (Figure 4). These oxy carotenoids were derived from acyclic or cyclic carotenes with a polyene system of at least 11 double bonds, and were synthesized as illustrated in Figure 1. Apart from the pathways to β-carotene, neurosporaxanthin, and astaxanthin (see Section 2), participating genes have not been identified yet and the enzymes involved in their synthesis are not known. Nevertheless, conceivable reaction mechanisms for the insertion of oxy groups into carotenoid molecules can be anticipated in analogy to those identified in bacterial carotenogenesis.

**Figure 5:**
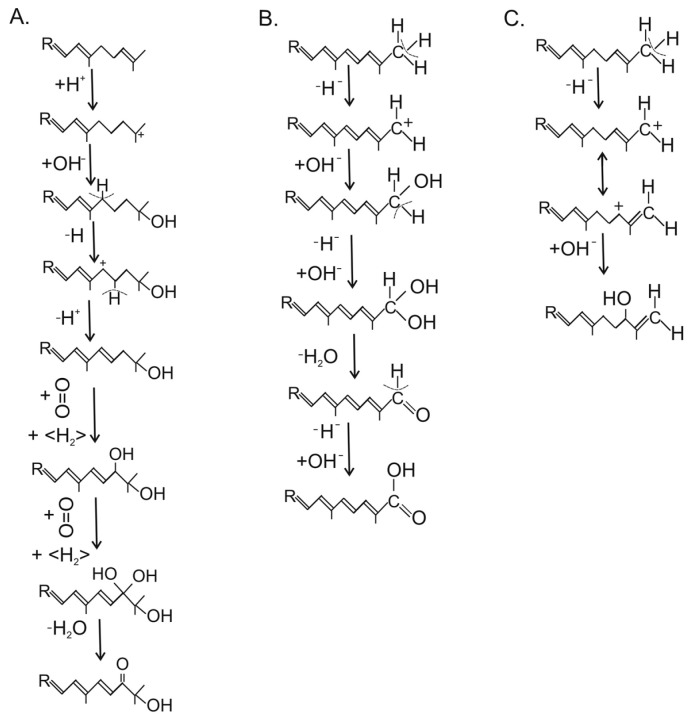
Proposed reaction mechanisms for the modification of acyclic end groups of fungal carotenoids leading to the formation of a 3′4′-didehydro-1′,2′-dihydro-1′-hydroxy-2′-one ψ-end group (**A**), carboxylic acid (**B**) and allylic 1′,2′-dihydro-1′,16′-didehydro 2′-ol (**C**). R indicates the carotenoid residue from six ionone terpenoid building blocks.

The C4/C4′-diketo β-carotene derivative canthaxanthin was reported for only one genus, the basidiomycete *Cantharellus* [51]. Three different reaction types for ketolation occurred in other species. The keto groups may originate from one of them. They include a P450 oxygenase Asy as in *X. dendrorhous*, or as in different bacteria, either a dioxygenase CrtW [58] or a CrtI-related ketolase CrtO forming an allylic carbocation by hydride transfer which reacts with a hydroxyl ion [59]. Finally, the keto group results from a second hydroxylation reaction including water elimination. Phillipsiaxanthin and 2′-didehydroplectaniaxanthin of Ascomycota and Basidiomycota share the same C1′-hydroxy and C2′-keto groups at the acyclic ends of the molecules (Figure 4, boxed region). Similarly to other hydroxylated fungal carotenoids, a certain amount was esterified with fatty acids. Their substitution pattern resembled the 3,4-didehydro acyclic end of demethylspheroidenone from the bacterium *Rhodobaca bogoriensis* grown under aerobic conditions [60] and may originate from similar reactions: The 1-OH group in this and other purple non-sulphur bacteria were formed by water addition to the C1/C2 double bond catalyzed by the *crtC* gene product (Figure 5a). This involved formation of a carbocation at C1 by protonation of this double bond, followed by its reaction with a hydroxyl ion [61]. In the following steps, a double bond was inserted at C3/C4 by CrtI-related CrtD [62], and the allylic C2 was ketolated by the monooxygenase CrtA in two hydroxylation steps with water elimination [63], as shown in Figure 5a. In plectaniaxanthin, this C2 carried a hydroxy instead of the keto group (Figure 4) due to a missing second step, indicating that this carotenoid is the precursor of 2′-didehydroplectaniaxanthin. It is difficult to decide whether torulene or γ-carotene was the original substrate for the hydroxylations. In the latter case, the biosynthesis of plectaniaxanthin could resemble its formation in the pathway to myxol in cyanobacteria [64]. Torularhodin, which is present in *Cookeina sulcipes* of Ascomycota and basidiomycetous red yeasts (Table 1), is an oxidation product of torulene with a C16′ carboxylic group (Figure 4). Unlike the carboxylic group of neurosporaxanthin, in which the keto moiety is formed by a cleavage oxygenase, a different reaction must be involved in torula-rhodin formation. An alternative method for generation of a carboxylic group at a terminal C-atom is known for 4,4′-diapolycopen-4-oate from *Bacillus* species [65]. The products of two *crtI*-related genes, *crtNb* and *crtNc*, first catalyzed the formation of a keto group by double hydroxylation and water elimination, followed by insertion of a hydroxy group (Figure 5b). Another C2′-hydroxy carotenoid, aleuriaxanthin from *Aleuria aurantia* (Table 1), is unique due to its terminal C16′ methylene group (Figure 4). The most likely reaction mechanism for its formation from γ-carotene starts with hydride transfer from C16′ to a cofactor, followed by a mesomeric shift of the C1′/C2′ double bond to C1′/C16′ (Figure 5c). Finally, the resulting C2′ carbocation reacted with a hydroxyl anion.

Most of the suggested reactions for oxygenation of the carotenoid skeleton in fungi were catalyzed by products of the *crtI*-gene family. In all cases, the first step was hydride transfer to oxidized FAD as a co-substrate. In the following step, the generated allylic carbocation was stabilized by different reactions, either proton loss forming a double bond or reaction with a hydroxyl ion to an alcohol, and in repetition of this step, to an aldehyde, and further on to an acid [66]. These mechanisms well resemble corresponding pathways in bacteria. Since the bacterial lineage is independent from the fungal one, analogous pathways may have evolved by convergent evolution. Since the CrtI phytoene desaturase was present in all carotenogenic fungi, *crtI*-derived genes could have diverged independently in fungi as well and acquired a modified novel function similar to bacteria. Alternatively, it is possible that genes encoding these bacterial enzymes have been acquired by horizontal transfer from bacteria. This process plays a significant role in fungal evolution [67].

## 4. Conversion of β-Carotene to Trisporic Acid in the Mucorales

**Figure 6:**
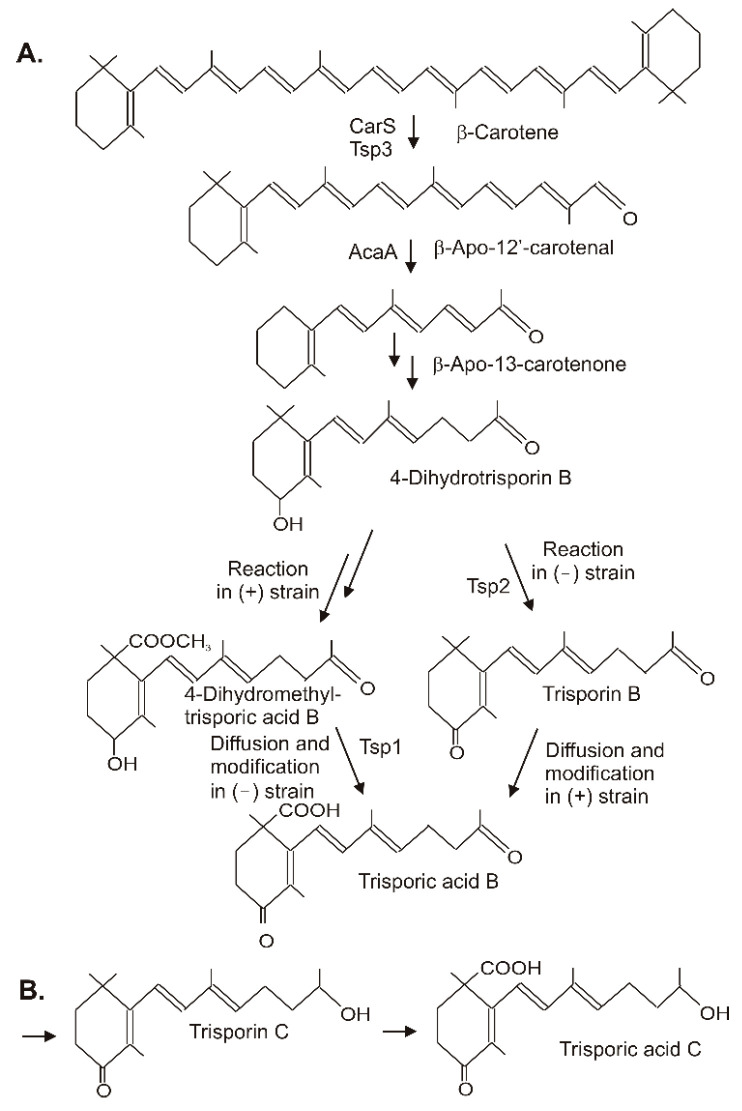
Synthesis of trisporic acids from β-carotene by interactions of different mating types in Mucoraceae. (**A**) Pathway to trisporic acid B; (**B**) Final step in trisporic acid C formation.

Trisporic acids were first recognized by their stimulation of β-carotene synthesis in fungi from the order Mucorales. However, the significant function is the recognition of opposite mating types in heterothallic species for the initiation of zygospore formation. A comprehensive review on the early developments, the significance of trisporic acids, the role in communication between strains, their structures, and their origin from β-carotene as precursor can be found in reference [68]. Different trisporic acids and their precursors with slight chemical modifications have been identified since then [69]. The structures of the B and C types of trisporic acids first identified from *B. trispora* [70] are shown in Figure 6 with the exemplified biosynthesis pathway to trisporic acid B [8]. It starts from β-carotene by two successive cleavage reactions [71]. The products of the gene *tsp3* from *Rhizopus oryzae* [72] or *carS* from *P. blakesleeanus* [73] cleave β-carotene at the C11′-C12′ double bond to β-apo-12′-carotenal in the initial reaction. The next cleavage step at the C13–C14 double bond by AcaA [73] leads to β-apo-13-carotenone, the C18 backbone of trisporoids. The following reactions to 4-dihydrotrisporin involve 4-hydroxylation and the saturation of the C11–C12 double bond (Figure 6A). For both reactions, the mechanisms have not been defined yet. The hydroxylation of the ionone ring may proceed via one of the steps described for 4-ketolation to canthaxanthin in Section 3. A saturation reaction is very unusual for carotenoids or related compounds. This hydrogenation of C11-C12 shortens the polyene system and isolates the terminal keto group.

The residual pathway continuing with the metabolization of 4-dihydrotrisporin relies on chemical interactions between the two mating partners [74]. Individual steps were catalyzed exclusively either by the (+) or the (−) strain. For pathway continuation, the reaction products from one strain must reach the other by diffusion. This metabolite exchange was necessary for the completion of the pathway to the trisporic acid end product. A 4-dihydrotrisporate dehydrogenase encoded by *tsp2* was active in the (−) strain in the formation of trisporin [75]. It was upregulated in the (−) strain upon mating, but not in the (+) strain. Alternatively, the (+) strain exclusively converts 4-dihydrotrisporin to the C15 carboxylic acid, and further on to its methyl ester in *B. trispora* [76] (Figure 6). Obviously, this ester was transferred to the (−) mating partner where it was oxidized at C4 by NADP-dependent 4-dihydromethyltrisporate aldo/keto reductase, which was isolated from *Mucor mucedo* as the product of the *tsp1* gene [77], and de-esterified to trisporic acid by a (−) strain-specific esterase [78]. In the (+) strain, trisporic acid finally accumulated from carboxylation of trisporin, which was taken up by diffusion from the (−) strain. The carboxylation reaction of the trisporins was not characterized. A possible mechanism may be similar to the carboxylation of a terminal C-atom in the synthesis of torularhodin (Section 3). In the closely related pathway to trisporic acid D, C4 was further oxidized to a keto group, whereas the keto group at C13 was reduced (Figure 6B). The trisporic acids exert a positive feedback on their synthesis and on the carotenoid pathway by transcriptional up-regulation of the pathway genes [69].

## 5. High-Yield Carotenoid Production

**Figure 7:**
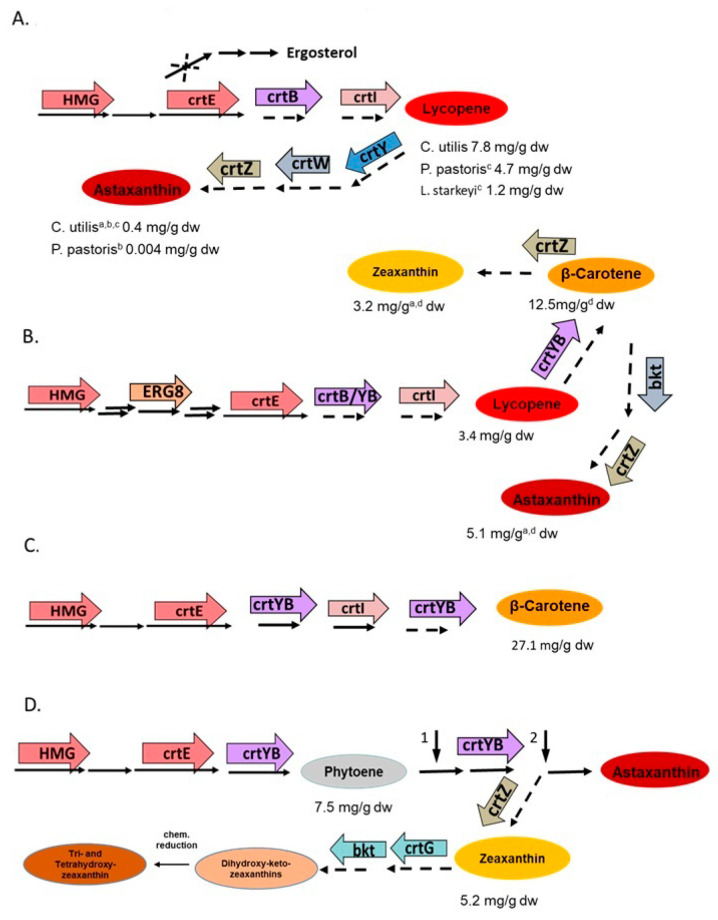
Selected examples of genetic engineering of carotenoids with highest yields in flask cultures on basic medium of different non-conventional yeasts by expression of existing genes, or genes for novel pathway (indicated as dotted arrows). (**A**) *Lipomyces starkeyi* [80] or *Candida utilis* [81,82] or *Pichia pastoris* [83,84]. (**B**) *Yarrowia lipolytica* [85,86,87,88]. (**C**) *Rhodotorula utilis* [89]. (**D**) *Xanthophyllomyces dendrorhous* [90,91,92]. The chosen uniform assignment of genes of same function also stands for corresponding fungal genes, as listed in Figure 1. Vertical arrow 1 marks inactivation of the phytoene desaturase gene; vertical arrow 2, inactivation of the astaxanthin synthase gene. ^a^ without gene *HMG*; ^b^ without *crtE*; ^c^ without squalene synthase inactivation; ^d^ without *ERG8* gene.

Fungi are promising candidates for the production of carotenoids. These pigments are of commercial interest: β-carotene, as provitamin A; astaxanthin, as an antioxidant; and zeaxanthin together with lutein, as protectants of the retina in the eye [79]. In addition, substantial amounts of astaxanthin and β-carotene were applied as feed additives. Carotenogenic fungi can be used best as a carotenoid source after mutagenesis and selection for over-producing strains. Alternatively, non-carotenogenic fungi are appropriate hosts for the design and engineering of carotenoid pathways [80]. Modification examples are outlined in Figure 7, with a focus on the initial highest carotenoid yields under laboratory conditions for comparison of the biosynthesis potential of the transformants. These strategies are advantageous for the generation of strains which are able to accumulate carotenoids comparable other organisms.

### 5.1. Classical Strain Development by Mutagenesis and Selection for Enhanced Carotenoid Production

Mutagenesis for alteration of the carotenoid pathway including the generation of high-yield strains was first successfully carried out with *P. blakesleeanus* [93]. The carS-type mutants obtained by chemical mutagenesis accumulated up to 5.1 mg/g dw, which is more than 100-fold the amount of β-carotene in the wild type. A similar type of mutation was obtained with *F. fujikuroi*, which resulted in increased neurosporaxanthin formation [94]. Considerable attempts were made to mutagenize *X. denrorhous* for enhanced astaxanthin production. This involved mutagenesis by chemicals, low-energy ion beam, or low-dose gamma irradiation [95]. Several rounds of nitrosoguanindin treatment resulted in a mutant with the high astaxanthin content of 1.6 mg/g dw [96]. An established industrial process for β-carotene production uses *B. trispora* and involves co-cultivation of (+) and (−) strains. Highest yields of 39 mg/g dw were obtained with improved mutants or intersexual heterokaryons with nuclei of both mating types [97]. The same strains can be used alternatively for the production of lycopene by application of a lycopene cyclase inhibitor. β-Carotene production with *B. trispora* is at the moment the only large-scale industrial process for fungal carotenoids. This carotenoid was recovered by extraction and supplied in a crystalline form as a colorant or as provitamin A [79].

### 5.2. Metabolic Engineering of Carotenoid Pathways in Non-Conventional Yeasts

Different non-carotenogenic yeasts were genetically engineered for carotenoid biosynthesis [80]. This started with the transformation of *Candida utilis* with bacterial genes to establish the pathway to astaxanthin. The genes used were *crtB* for phytoene synthesis, *crtI* for phytoene desaturation to lycopene, *crtY* for cyclisation to β-carotene, and *crtW* together with *crtZ* for β-carotene ketolation and hydroxylation to astaxanthin (Figure 7A), which yielded 0.4 mg/g dry weight of astaxanthin [81]. The next-generation of recombinant *C. utilis* strains were modified in key metabolic steps by over expression of the gene for HMG-CoA reductase, *HMG*, which is the limiting enzyme for the entire terpenoid pathway [82]. In addition, one of two alleles of the squalene synthase gene was inactivated together with over expression of the geranylgeranyl pyrophosphate synthase gene *crtE* to re-direct metabolite flow from the sterol pathway into carotenoids (Figure 7A). This resulted in an increase of lycopene to 7.8 mg/g dw. A similar genetic modification of the pathway to lycopene without inactivation of the squalene synthase gene was carried out in a similar way with *Lipomyces starkeyi* [80] and *Pichia pastoris* [83], reaching a lycopene concentration of 1.2 and 4.7 mg/g dw, respectively. With the same genes used for *C. utilis*, the carotenoid pathway was also extended to astaxanthin in *P. pastoris*, but only 0.004 mg/g dw were obtained [84].

Considerable progress has been made with metabolic engineering of *Yarrowia lipolytica*, including the establishment of carotenoid pathways [98]. First, application of CRISPR-Cas9 mediated integration of the bacterial carotenogenic genes: *crtB* for phytoene synthesis, and *crtI* for lycopene formation and enhancement of the terpenoid pathway by additional expression of the genes of *HMG*, phosphomevalonate kinate *ERG8*, and *crtE* led to the formation of 3.4 mg/g dw lycopene [85]. Using a similar set of fungal genes except *ERG8* and replacement of the phytoene synthase gene *crtB* by the bifunctional phytoene synthase/lycopene cyclase gene *crtYB*, all conventionally genome-integrated, the pathway was extended to β-carotene (Figure 7B), and a concentration of 12.5 mg/g dw was obtained [86]. Additional integration of multi copies of a bacterial β-carotene hydroxylase gene *crtZ* extended the pathway to zeaxanthin reaching 3.2 mg/g dw of this carotenoid [87]. There is only one step left in the pathway to astaxanthin, which is carried out by a ketolase. By replacement of the bacterial *crtZ* by an algal hydroxylase gene and the use of the algal β-carotene ketolase gene *bkt*, about half the synthesized carotenoids were converted to astaxanthin at a yield of 5.1 mg/g dw, as calculated from fermenter cultures [88].

Carotenogenic yeasts offer the advantage of an already existing terpenoid pathway, which is directed towards carotenoid biosynthesis and a carotenoid storage system. A β-carotene accumulating strain of *Rhodotorula glutinis* with enhanced pathway by over-expression of limiting and pathway genes including *crtYB* (Figure 7C) resulted in a β-carotene yield of 27.1 mg/g dw [89]. Of importance for the final step to β-carotene was the *crtYB* gene. It favors the formation of bicyclic β-carotene over monocyclic γ-carotene, which is part of the competing pathway to torularhodin in *R. glutinis* [57]. A systematic genetic approach was accomplished to intervene with the astaxanthin biosynthesis pathway of *X. denrorhous* [99]. Starting from a high-yield astaxanthin mutant, the limiting steps were overcome by additional expression of the *HMG*, *crtI*, and *crtYB* genes (Figure 7D). For more efficient β-carotene conversion to astaxanthin, transformation with additional copies of the astaxanthin synthase gene *asy* was effective. This led to a strain with 9.0 mg/dw astaxanthin [90]. The same engineering steps were applied to accumulate the intermediate phytoene by inactivation of the endogenous phytoene desaturase gene *crtI* (Figure 7D, arrow 1), yielding 7.5 mg/g dw phytoene [91]. For the synthesis of zeaxanthin (3,3′-dihydroxy-β-carotene), which is not part of the carotenoid pathway of *X. denrorhous* nor of any other fungus, *asy* was inactivated (Figure 7D, arrow 2) and the accumulated β-carotene converted to zeaxanthin with a bacterial β-carotene hydroxylase gene *crtZ* [92]. It was important to insert several copies of *crtZ* into the genome for substantial metabolization of β-carotene to zeaxanthin reaching up to 5.2 mg/g dw. *X. denrorhous* is also useful for building novel structures of carotenoid. By a combination of bacterial genes encoding β-carotene 2-hydroxylase, 3-hydroxylase, and 4-ketolase, the novel structures 2,2′-dihydroxy-4,4′-diketo-zeaxanthin, and 2,2′-dihydroxy-4-monoketo-zeaxanthin were generated [100]. For further processing, both carotenoids were chemically reduced, resulting in 2,4,2′4′-tetrahydroxy-zeaxanthin and 2,4,2′-trihydroxy-zeaxanthin. The pathway engineering leading to these multioxy carotenoids can be regarded as a proof-of-concept demonstrating *X. denrorhous* as a synthesis platform was applicable for the biosynthesis of novel structures.

The carotenoid concentrations of the transformants in Figure 7 were obtained under routine laboratory conditions. Starting with a lipid overproducing strain of *Y. lipolytica* for transformation [101], optimized media and fed-batch fermentation procedures can increase the carotenoid concentrations considerably. For example, 16 mg/g dw of lycopene [102] and 90 mg/g dw of β-carotene [101] were reached in fermenter cultures of engineered *Y. lipolytica*, as well as 10.0 mg/g dw phytoene [91] in fermenter culture of engineered *X. denrorhous*. Many yeast are able to grow on agricultural waste materials. For example, enhanced zeaxanthin synthesis was obtained with engineered *X. denrorhous* growing on wheat straw hemicellulose hydrolysate [92]. Furthermore, *Y. lipolytica* was improved by metabolic engineering to utilize of a wide range of substrates [103].

### 5.3. Pathway Engineering of Saccharomyces Cerevisiae for High-Yield Carotenoid Formation

*Saccharomyces cerevisiae* is an advanced system for metabolic pathway construction. Therefore, this yeast is not only a prominent system for establishing carotenoid pathways with similar genes used for other yeasts shown in Figure 7, but is also suitable to engineer around this pathway in order to boost the yields of the desired carotenoid. These combined approaches include modifications of the central metabolism for acetyl-CoA provision and modification of the lipid metabolism for increased carotenoid storage capacity [104].

In addition to the establishment of the lycopene pathway and the search for best possible gene combinations [105], the central metabolisms of *S. cerevisiae* were engineered to intensify the conversion of ethanol into acetyl-CoA [106,107] and to provide for a better NADPH supply [105,106]. Another target was the lipid metabolism. By overexpression of three genes of the pathway to triacylglycerols, lipid production was increased with a higher share of monoenic fatty acids due to a simultaneous overexpression of the desaturase gene *OLE1* [106]. In combination with the inactivation of a regulator of lipid droplet size, the improved storage capacity corresponded to a higher accumulation of lycopene in fed-batch fermentation to a titer of 2.37 g/L and a yield of 73.3 mg/g dw.

The development of a β-carotene producing *S. cerevisiae* is less advanced. A transformant carrying the genes from *X. dendrorhous* for β-carotene synthesis [108] was subjected to adaptive laboratory evolution in combination with oxidative stress and selection of hyper-producing mutants [109]. By sequencing their genomes, several mutations conferring H_2_O_2_ tolerance were identified, which were also accountable for higher β-carotene synthesis of up to 16.4 mg/g dw. A straightforward strategy for enhancing the carotenoid pathway is the adaptation of *S. cerevisiae* substrate utilization. Transformation with extracellular and cell-bound lipases enabled a β-carotene producing strain to grow on olive oil exclusively as a way to use olive mill waste as a substrate [110]. Supplementation of growth media with olive oil increased the lipid content of this strain. These lipids were able to provide a substantial supply of acetyl-CoA for carotenoid synthesis, which resulted in a β-carotene accumulation of 46.5 mg/g dw and a titer of 477.9 mg/L.

For the synthesis of astaxanthin from β-carotene, additional ketolation and hydroxylation steps are necessary. After screening for optimum ketolase and hydroxylase gene combinations, random mutagenesis resulted in a library including several mutants with increased astaxanthin formation [111]. For one of them with mutated *CCS1* gene, transcriptional profiles indicated an impact on sterol biosynthesis. Higher formation of sterols may also suggest better metabolite supply for carotenogensis. In fermenter cultures of this mutant, the astaxanthin yield was 13.8 mg/g dw, accounting for a remarkable 89.4% of total carotenoids, and a titer of 0.22 mg/L.

## 6. Conclusions

Fungi are of biotechnological interest as biotechnological production platforms for commercially important metabolites. Furthermore, they have a high potential for genetic pathway engineering. In fungi, special carotenoids of unique structures exist. For some of them, the biosynthesis pathways have been established by mutant analysis and functionality studies of the pathway genes. However, for the majority of fungal carotenoids, very little is known about the reaction sequences, the enzymes, and the genes involved in their biosynthesis. By comparison to well-known bacterial carotenogenic reactions, mechanisms for the insertion of oxy groups into carotenoid molecules were proposed. They may give a clue on the expected types of genes involved in fungal carotenogenesis and may be helpful for their identification in the future.

Metabolic engineering of carotenoid pathway especially in yeasts, either carotenogenic or non-carotenogenic, has made considerable progress in recent years. Combination of mutagenesis, adaptive laboratory evolution of carotenogenic lines, genetic engineering of the central carbon metabolism to enhance acetyl-CoA supply, and modification of the triacylglycerol lipid pathway to improve carotenoid storage capacity led to the construction of high-yield carotenoid-producing strains. Apart from mass produced β-carotene with *B. trispora* which already made it into the market, astaxanthin from engineered *X. dendrorhous*, with a content of almost 1% of cell mass in non-optimized laboratory cultures [90], can compete with astaxanthin from *Haematococcus* species, especially as it is accumulating in a non-esterified form [79]. Metabolic engineering of *Y. lipolytica* [87], and particularly *X. dendrorhous* [92], resulted in synthesis of the rare carotenoid zeaxanthin matching the concentrations of the high-yield mutant of *Dunaliella salina* [112].

Microorganisms such as yeasts offer the advantage of a controlled fermentation process. Development of advancing conditions for customized fermentation and application of selected substrates can make these transgenic yeasts attractive for commercial utilization. This includes production of carotenoids by growing on agroindustrial waste materials. This recycling process may work with the original strains or with those further engineered for the utilization of a specific substrate.

## Figures and Tables

**Table 1:** Fungal carotenoids and their direct carotene precursors in selected species.

Carotenoids	Selected Species	References
Lycopene ---> γ-Carotene	**Chytridiomycota:**	[2]
	*Cladochytrium replicatum*	
	Blastocladiomycota	
	*Allomyces arbuscula*	
Lycopene ---> β-Carotene	**Mucoromycotina:**	[1]
	*Blakeslea trispora*	
	*Phycomyces blakesleanus*	
	**Ascomycota:**	[48]
	*Protomyces inundates*	
	**Basidiomycota:**	[49]
	*Tremella mesenterica*	[50]
	*Gymnosporangium juniperi-virginianae*	
Lycopene ---> Phillipsiaxanthin	**Ascomycota:**	[2]
	*Phillipsia carminea*	
β-Carotene ---> Canthaxanthin	**Basidiomycota:**	[51]
	*Cantharellus* species	
β-Carotene ---> Astaxanthin	**Basidiomycota:**	[38]
	*Xanthophyllomyces dendrorhous*	
γ-Carotene ---> Aleuriaxanthin	**Ascomycota:**	
	*Aleuria aurantiaca*	[52]
	*Scutellina umbrarum*	[45]
γ-Carotene and/or torulene ---> Neurosporaxanthin	**Ascomycota:**	
	*Fusarium* species	[23]
	*Neurospora crassa*	[30]
γ-Carotene and/or torulene ---> Plectaniaxanthin and 2′-didehyroplectaniaxanthin	**Ascomycota:**	
	*Plectania coccinea*	[53]
	*Sarcoscypha coccinea*	[54]
	**Basidiomycota:**	
	*Cryptococcus laurentii*	[55]
	*Dioszegia species*	[46]
Torulene ---> Torularhodin	**Ascomycota:**	[56]
	*Cookeina sulcipes*	
	**Basidiomycota:**	[57]
	*Cystofilobasidium* species	[47]
	*Rhodotorula glutinis*	

For structures, see Figure 4; for early steps, see Figure 1.

## Abbreviations

CoA	coenzyme A
dw	dry weight
HMG	hydroxymethylglutaryl
NADPH	nicotinamide adenine dinucleotide phosphate
